# Immunotherapy of Hepatocellular Carcinoma with Magnetic PD-1 Peptide-Imprinted Polymer Nanocomposite and Natural Killer Cells

**DOI:** 10.3390/biom9110651

**Published:** 2019-10-25

**Authors:** Mei-Hwa Lee, Kai-Hsi Liu, James L. Thomas, Jyun-Ren Chen, Hung-Yin Lin

**Affiliations:** 1 Department of Materials Science and Engineering, I-Shou University, Kaohsiung 84001, Taiwan; meihwalee@ntu.edu.tw; 2 Department of Internal Medicine, Division of Cardiology, Zuoying Branch of Kaohsiung Armed Forces General Hospital, Kaohsiung 813, Taiwan; liukaihsi@gmail.com; 3 Department of Chemical and Materials Engineering, National University of Kaohsiung, Kaohsiung 81148, Taiwan; 4a140109@stust.edu.tw; 4 Department of Physics and Astronomy, University of New Mexico, Albuquerque, NM 87131, USA; jthomas@unm.edu

**Keywords:** immunotherapy, human hepatoma cells, programmed cell death protein 1 (PD-1), magnetic nanoparticles, peptide-imprinted polymer, natural killer cells

## Abstract

Programmed cell death protein 1 (PD-1) is a biomarker on the surface of cells with a role in promoting self-tolerance by suppressing the inflammatory activity of T cells. In this work, one peptide of PD-1 was used as the template for molecular imprinting to form magnetic peptide-imprinted poly(ethylene-*co*-vinyl alcohol) composite nanoparticles (MPIP NPs). The nanoparticles were characterized by dynamic light scattering (DLS), high-performance liquid chromatography (HPLC), Brunauer–Emmett–Teller (BET) analysis, and superconducting quantum interference device (SQUID) analysis. Natural killer 92 (NK-92) cells were added to these composite nanoparticles and then incubated with human hepatoma (HepG2) cells. The viability and the apoptosis pathway of HepG2 were then studied using cell counting kit-8 (CCK8) and quantitative real-time polymerase chain reaction (qRT-PCR), respectively. These nanoparticles were found to significantly enhance the activity of natural killer cells toward HepG2 cells by increasing the expression of nuclear factor kappa B (NF-κB), caspase 8, and especially caspase 3.

## 1. Introduction

Programmed cell death protein 1 (PD-1), which was discovered by Honjo in 1992 [1], is expressed on the surface of T cells and plays a role in promoting the self-tolerance of those cells by suppressing their inflammatory activity. The clinical use of PD-1 as a target in the treatment of patients with several types of cancer began in 2012 [2,3]. The cancers thus treated include lung cancer, renal cancer, lymphoma, and melanoma, as reviewed by Guan et al. [3]. Trials of this treatment against these cancers used immune checkpoint therapy with various targets on the cellular surface, such as cytotoxic-lymphocyte antigen-4 (CTLA-4) and programmed cell death protein-1 or its ligand (PD-1/L1) [3]. Many anti-PD-1 and anti-PD-L1 agents were developed, and some of them (such as pembrolizumab, nivolumab, and atezolizumab) were approved by the Food and Drug Administration (FDA) for melanoma, non-small-cell lung cancer, renal cell carcinoma, Hodgkin lymphoma, and metastatic uroepithelial carcinoma [3]. Combination therapies, such as (anti-PD-1/anti-PD-L1) [4] or (anti-CTLA-4/anti-PD-1) [5], also underwent human trials.

Hepatocellular carcinoma (HCC) is a leading cause of cancer-related morbidity and mortality [6], especially in Asia. The immunological landscape and immunotherapy for HCC, which were reviewed by Prieto et al. [7], suggest that novel treatment methods are urgently needed. In HCC, the liver is enriched with various innate immune cells [8], among which natural killer (NK) cells are important in host defense and maintaining immune balance [9]. The expression of PD-L1 was found in seven human HCC cell lines and 240 randomly selected HCC patients who underwent surgery, and the overexpression of PD-L1 is significantly associated with tumor aggressiveness and postoperative recurrence [10]. Liver NK cells can produce interferon-γ and tumor necrosis factor-α following the recognition of induced-self proteins on cancer cells by the natural killer group 2 member D (NKG2D) receptor [11]. While PD-L1 expression allows cancer cells to evade immune surveillance by suppressing T-cell-mediated immunity [3], natural killer (NK) cells can also contribute to immunotherapy mediated by a PD-1/PD-L1 blockade; cancers with low major histocompatibility complex (MHC) expression are responsive to PD1 blockade via a strong NK cell response [12]. Four clinical trials in which the NK-92 cell line was used, rather than blood NK cells, were reported [13]. Those phase I studies used pre-infusion irradiation of the (malignant) NK-92 cells to abrogate proliferation, rendering infusions safe with no severe unexpected side effects [13].

In 2007, Langer’s group reported nanocarriers as an emerging platform for cancer therapy [14]. Nanocarriers are typically liposomes, inorganic particles, metallic shells, amphipathic molecules, dendrimers, or carbon nanotubes; these may incorporate biodegradable polymers/chemotherapeutics/surface functionalities/spacer groups/agents to promote a long circulation time, as well as targeting molecules (aptamers, antibodies, or their fragments) [14]. The safe delivery of nanoparticles [15], their design [16], and their applications in cancer therapy [17] were extensively reviewed, including the subjects of molecularly targeted immunotherapy, nanoparticle delivery of immunotherapy agents, systemic gene delivery of immunomodulators to tumors, and the use of immune cells as drug carriers and targeting immunotherapy to immune cells [15].

Molecularly imprinted polymers (MIPs) are synthesized with cavities with size and structure complementary to the template molecules. Upon removal of the template, they can be used as artificial antibodies [18]. Conventionally, the template molecule that is used in the imprinting of proteins is the whole protein, thereby providing high selectivity and sensitivity. Potential disadvantages of whole protein templates may include solubility limitations and price [19]. Thus, inexpensive proteins such as albumin [20], lysozyme [21], or hemoglobin were employed in model systems. Recently, useful peptide epitopes of proteins were found via molecular simulation [22] or empirical methods [23], and used as templates in molecular imprinting. Peptide-imprinted polymers were characterized and found to achieve both high capacity and selectivity [23]. Peptides that contain six to 14 amino acids are typically selected as epitope templates for imprinting [22]. Currently, however, there are no general rules for optimal peptide selection.

Finding epitopes for enzymes, cell signaling and ligand-binding proteins, and structural proteins may involve different considerations, such as the location of the active sites of the enzyme, or the exposed structures for ligand binding or for structural proteins. Also, the solubility and interfacial properties of epitopes are important in the preparation of MIP nanoparticles (NPs) [20,24]. Our previous work demonstrated the preparation and the characterization of MIPs and cancer-related applications [25,26]; for example, a small molecule (orcinol) was templated onto MIP NPs for the extraction of an important bioactive molecule (resveratrol) from Chinese herb extract for oral cancer treatments [25], and a thymine-imprinted polymeric NP was used to promote the expression of p53 in hepatoma cells [26]. In this work, a peptide derived from PD-1 was identified for molecular imprinting. Magnetic peptide-imprinted polymer nanoparticles (MPIP NPs) were formed by the phase separation of poly(ethylene-*co*-vinyl alcohol), EVAL, and then characterized by dynamic light scattering (DLS) and surface area analysis. The isothermal adsorption and capacity of MPIP NPs were measured using HPLC. Finally, hepatoma (HepG2) cells were treated with PIP NPs and natural killer (NK-92) cells. The immune pathway of HepG2 cells with PIP NPs was investigated using quantitative reverse transcription-polymerase chain reaction (qRT-PCR).

## 2. Materials and Methods

### 2.1. Reagents

Peptide AISLHPKAKIEES (Peptide A) of mPD-1 (murine PD-1) was designed and ordered from Yao-Hong Biotechnology Inc. (HPLC grade, New Taipei City, Taiwan). PD-1 and PD-L1 interact through the conserved front and side of their immunoglobulin (Ig) variable (IgV) domains, as determined from the crystal structure of murine PD-1 in complex with human PD-L1 [27]; thus, the use of mPD-1 is suitable for this proof-of-concept study. Poly(ethylene-*co*-vinyl alcohol), EVAL, with ethylene 27, 32, 38, and 44 mol.% (product no. 414077, 414093, 414085, 414107) and RT-PCR primers were from Sigma-Aldrich Co. (St. Louis, MO, USA). Iron(III) chloride 6-hydrate (97%), iron(II) sulfate 7-hydrate (99.0%), and dimethyl sulfoxide (DMSO, product # 161954) were from Panreac (Barcelona, Spain). DMSO was used as the solvent to dissolve EVAL polymer particles in a concentration of 1 wt.%. Absolute ethyl alcohol was from J. T. Baker (ACS grade, Phillipsburg, NJ, USA). All chemicals were used as received unless otherwise mentioned.

### 2.2. Synthesis and Formation of Magnetic Molecularly Imprinted Polymer Composite Nanoparticles

The synthesis of magnetic peptide-imprinted EVAL composite nanoparticles (MPIP NPs) included several steps. Magnetic nanoparticles, synthesized by co-precipitation of a mixture of iron(III) chloride 6-hydrate and iron(II) sulfate 7-hydrate by sodium hydroxide, were repeatedly washed while adsorbed on a magnetic plate. The magnetic nanoparticles were washed three times with deionized water, and then dispersed using ultrasonication for 30 s. The peptide was dissolved in an appropriate volume of deionized water, yielding final concentrations of 0.2, 2, 20, 100, and 200 μg/mL. Then, 250 μL of DMSO solution was added to the same volume of peptide solution to form a clear EVAL solution, and 10 mg of composite particles were then added. Next, 0.5 mL of EVAL solution was dispersed in 10 mL of deionized water, the template molecule was removed by washing in 10 mL of deionized water 15 min for three times, separating the MPIPs magnetically after each wash. The non-imprinted polymers (NIPs) were prepared identically without peptide addition.

### 2.3. Characterization of Magnetic Molecularly Imprinted Polymer Composite Nanoparticles

Magnetic (MNP), peptide-imprinted (MPIP) and non-imprinted (MNIP) EVAL composite particles were monitored using a particle sizer (90Plus, Brookhaven Instruments Co., Holtsville, NY, USA). The dynamic light scattering (DLS) measurement of the particle size distribution was done at 25 °C with 3 min of data collection at a 90° detection angle. The magnetization of magnetic, peptide-imprinted polymeric nanoparticles before and after template removal was monitored with a magnetic property measurement MPMS XL-7 system (Quantum Design, San Diego, CA, USA) at 298 K in ±15,000 Gauss.

The adsorption of peptide on nanoparticles was examined with a high-pressure liquid chromatography (HPLC) system. A peptide solution (1 mg/mL) in water was diluted to various concentrations for calibration. The solutions were passed through a 0.22-μm poly(vinylidene fluoride), PVDF, syringe filter (Advangene Consumables Inc., Lake Bluff, IL, USA) and then injected into a high-pressure liquid chromatography (HPLC) system for analysis of peptide. The separation was performed on a Kromasil 100-5 C18 column (5 μm particle size, pore size 100 Å, 25 cm × 4.6 mm I.D., Sigma-Aldrich, St. Louis, MO, USA). The sample (20 μL) was eluted with a mobile phase composed of 0.1% trifluoroacetic acid (TFA) in water (solvent A) and 0.1% TFA in acetonitrile (solvent B). The gradient profile of peptide A was as follows: 0–0.1 min, 12% A, 88% B; 0.1–25 min, 37% A, 63% B; 25.01–30 min, 100% A. The flow rate and detection wavelength were set to 1.0 mL/min and 220 nm, respectively. The retention time was 6.19 min for peptide A.

### 2.4. Cytotoxicity Test of HepG2 Cells with Magnetic Molecularly Imprinted Composite Nanoparticles and NK-92 Cells.

#### 2.4.1. MTT Assay

HepG2 (Human hepatoblastoma cells, BCRC # 60364) cells were cultured in a 90% mixture of 1:1 Dulbecco’s modified Eagle’s medium (DMEM) and Ham’s F12 medium with 10% heat-inactivated FBS (fetal bovine serum), supplemented with 0.4 mg/mL G418 (Geneticin) at 37 °C and 5% CO_2_. For the cytotoxicity experiments, about 10 μL of 7.5 × 10^5^ HepG2 cells/mL and 90 μL of culture medium per well (i.e., 7.5 × 10^3^ HepG2 cells per well) were seeded in 96-well culture plates and then incubated at 37 °C in 5% CO_2_ for 24 h. Various concentrations of nanoparticles were added to each well at 37 °C for 24 h. Twenty microliters of MTT (3-(4,5-dimethylthiazol-2-yl)-2,5diphenyl–tetrazolium bromide, a yellow tetrazole) solution in phosphate-buffered saline (PBS) was added to each well after 24 h, and then wells were incubated in 5% CO_2_ for 3 h at 37 °C. The solution was removed from each well. Then, 100 μL of dimethyl sulfoxide (DMSO) was added to each well, followed by incubation at 37 °C for 30 min in the dark. The absorbance of this colored solution could be quantified at a measuring wavelength of 570 nm using 690 nm as reference by an ELISA reader (CLARIOstar Reader, BMG Labtech, Offenburg, Germany). Effective adsorption was obtained by subtracting the reference wavelength absorption (which corrects for turbidity/scattering) from the measured wavelength absorption. The percentage of viable cells was determined from the ratio of effective absorption of experimental cells to controls. Experiments were carried out in quadruplicate wells and repeated at least three times.

#### 2.4.2. Cell Counting Kit-8 (CCK-8) WST-8 Cell Proliferation Cytotoxicity Assay

NK-92 (Human natural killer cell, BCRC # 60414) cells were cultured in Roswell Park Memorial Institute (RPMI) 1640 with l-glutamine and 10% FBS at 37 °C and 5% CO_2_. The CCK-8 test (Sigma-Aldrich Chemical Co., St. Louis, MO, USA) was used as a rapid and sensitive method for toxicity of MNIPs, MNIPs with NK-92 cells, and MPIPs with NK-92 cells. Firstly, 200 μL of 2.5 × 10^5^ HepG2 cells/mL and 1.8 mL of culture medium per well (i.e., 5 × 10^4^ HepG2 cells/well) were seeded in 24-well culture plates and then incubated at 37 °C in 5% CO_2_ for 24 h. The synthesized MNIP or MPIP composite nanoparticles were mixed with NK-92 cells medium for 10 min. NK-92 cells with NIPs or MPIPs (0.5 mL) were added to HepG2 cells, and incubated at 37 °C in 5% CO_2_ for 24 hr. Particles were removed from wells, and 500 μL of CCK-8 solution was added before ELISA measurements. The absorption intensities were measured by an ELISA reader (CLARIOstar, BMG Labtech, Offenburg, Germany) at a wavelength of 450 nm (I_450_), and the reference absorption (I_ref_, to account for turbidity and scattering) was obtained at a wavelength of 650 nm. The cellular viability (%) was then calculated from the ratio of effective absorption (I_450_–I_ref_) of experimental cells to controls. CCK-8 solutions were removed, and 2 mL of medium was added to each well for the next round of the cellular viability test.

### 2.5. Gene Expression of HepG2 Cells Treated with Molecularly Imprinted Polymer Composite Nanoparticles

The sequences (5′–3′) of primers for *NF-κβ1*, *Caspase 8*, *Caspase 3*, and glyceraldehyde 3-phosphate dehydrogenase (*GAPDH*) were as follows: *NF-κβ1*, forward: CACAAGGAGACATGAAACAG, reverse: CCATGACAGCAAATCTCC; *Caspase 8*, forward: CTACAGGGTCATGCTCTATC, reverse: ATTTGGAGATTTCCTCTTGC; *Caspase 3*, forward: AAAGCACTGGAATGACATC, reverse: CGCATCAATTCCACAATTTC; *GAPDH*, forward: ACAGTTGCCATGTAGACC, reverse: TTTTTGGTTGAGCACAGG. The total RNA extraction from the HepG2 cells cultured one day after NP administration was purified using the KingFisher Total RNA Kit and the KingFisher mL magnetic particle processors, both from Thermo Scientific (Vantaa, Finland). The concentration of cellular RNA was quantified by determining the absorbance maximum at the wavelengths of 260 and 280 nm to optimum optical density between 1.6 and 2.0 in a UV/Vis spectrometer (Lambda 40, PerkinElmer, Wellesley, MA, USA). Complementary DNA was obtained following a Deoxy+ real-time 2× SYBR green RT-PCR kit (Yeastern Biotech Co., Ltd., Taiwan) protocol. The real-time PCR was then performed in a PikoReal real-time PCR system (Thermo Scientific, Vantaa, Finland). Relative gene expression was determined using the ΔΔCq method [28] and normalized to a reference gene (*GAPDH*) and to a control (HepG2).

### 2.6. Data Analysis

All experiments were carried out in triplicate, and data are expressed as means ± standard deviation. The cellular viability and gene expression data were analyzed with Student’s *t*-test. Statistical significance was set at a *p*-value of less than 0.05, and *p*-values < 0.005 were considered highly significant.

## 3. Results and Discussion

**Figure 1:**
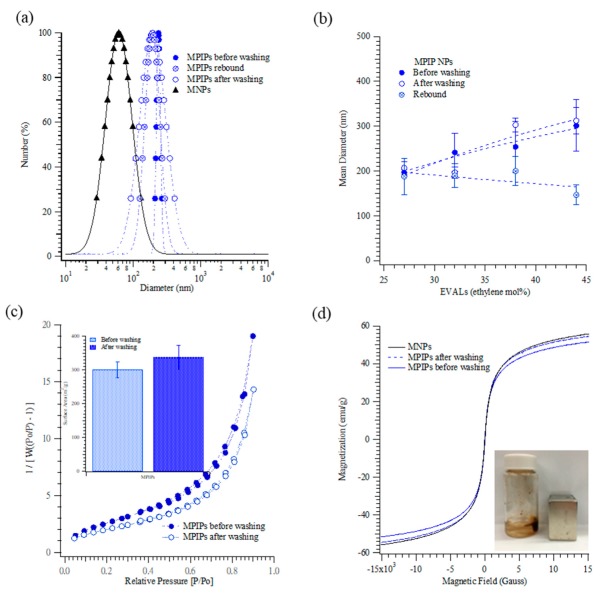
(**a**) Particle size distributions of magnetic nanoparticles (MNPs) (▲), as well as magnetic peptide-imprinted polymers (MPIPs) before (●) after (○) template removal, and rebound with template (⊕). (**b**) Mean diameters of MPIPs before (●) and after (○) template removal, and rebound with template (⊕) containing various ethylene concentrations (mol.%) of poly(ethylene-*co*-vinyl alcohol (EVAL). (**c**) Surface area of MPIPs before (●) and after (○) template removal measured by adsorption and desorption of nitrogen. (**d**) The magnetization of MNPs and MPIPs before and after template removal. Inset: MPIPs on the walls of a vial under magnetic field.

Figure 1 shows the characterization of MPIP NPs before the in vitro experiments. The DLS examination of NPs was a rapid process for measuring their size distribution and identifying their nanocomposite coating. Particle size distributions are shown in Figure 1a. The mean sizes of the magnetic nanoparticles, and peptide-imprinted nanoparticles (32 mol.% ethylene) before and after template removal were 61 ± 6, 241 ± 43, and 197 ± 12 nm, respectively; the size of the peptide-imprinted NPs decreased slightly to 189 ± 26.45 nm upon the rebinding of the peptide. Figure 1b displays the mean sizes of MPIP NPs that were prepared with various concentrations of ethylene (mol.%). Interestingly, increasing ethylene content caused the mean size of MPIP NPs to increase (from 207 ± 14 to 312 ± 29 nm, before template removal, for ethylene content from 27 to 44 mol.%), but caused the particles with rebound target to shrink slightly, from 188 ± 41 to 147 ± 22 nm. Thus, the rebinding target caused a much greater size contraction when the ethylene content was higher.

Surface area measurements included the adsorption and desorption of nitrogen and Brunauer–Emmett–Teller (BET) analysis, as presented in Figure 1c. The lack of a knee in the curves reflected the extremely weak adsorbate–adsorbent interactions between MPIPs and nitrogen. The specific areas under the curves before and after the MPIPs were washed were 301.4 ± 23.9 and 337.7 ± 35.4 m^2^/g, respectively.

**Figure 2:**
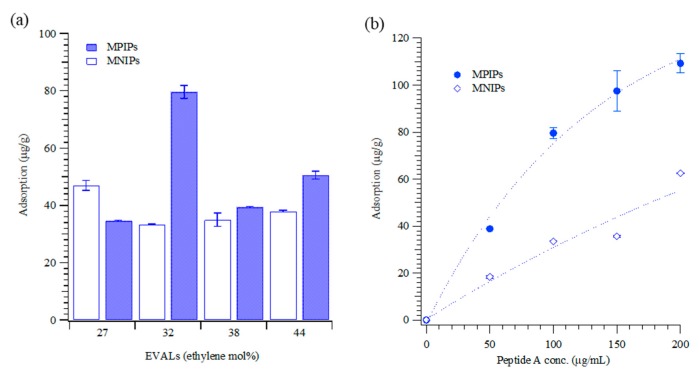
(**a**) Adsorption of peptide (100 μg/mL) on the MPIPs imprinted onto various ethylene concentrations (mol.%) of EVAL. (**b**) Isothermal adsorption of MPIPs (●) and NIPs (○) with various peptide concentrations.

The specific area after the MPIPs were washed was higher than before washing. The results reveal that target molecules (peptide A) were removed from the surface of MPIPs by washing, increasing their adsorption capacity. Figure 1d plots the magnetization curves of the MNP and PIP NPs before and after template removal. The saturated magnetization was approximately 52–56 emu/g, which may indicate that a few MNPs were present in a single MPIP NP. The removal of the template from the surface of NPs resulted in a small drop in magnetization. Figure 2 shows the adsorption peptides on the MPIP NPs. Figure 2a plots the capacities of MPIP and MNIP NPs that were prepared with various concentrations of ethylene from 27 to 44 mol.%. The highest rebinding capacity of MPIP NPs was achieved using EVAL that contained 32 mol.% ethylene. The imprinting effectiveness was defined as the ratio of peptide adsorption on MPIP to that on MNIP, and the results are presented in Table 1. The imprinting effectiveness of MPIPs with EVAL that contained 32 mol.% ethylene was 2.38, but other EVALs gave rather poor imprinting effectiveness, with nearly as much (or in one case, even more) peptide binding to non-imprinted controls as to imprinted NPs. MPIPs with 32 mol.% ethylene were, thus, employed in the cellular experiments. Figure 2b shows the titrations of MPIPs and MNIPs (32 mol.% ethylene) with peptide A.

**Figure 3:**
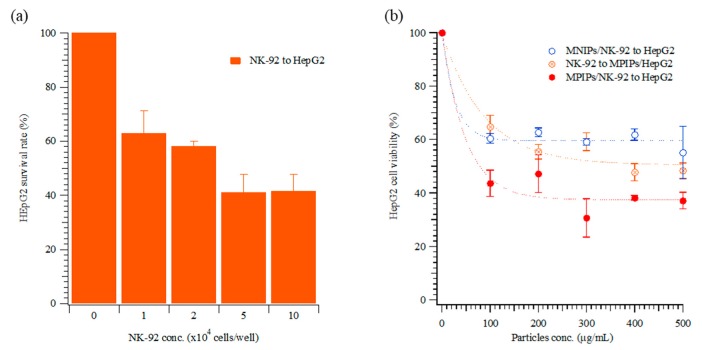
Cellular viability of HepG2 cells with various concentrations of (**a**) natural killer (NK)-92 cells, and of (**b**) MNIPs incubated with NK-92 cells, with both then applied to HepG2 cells (○), MPIPs incubated with HepG2 cells, followed by addition of NK-92 cells (⊗), and MPIPs incubated with NK-92 cells, with both then applied to HepG2 cells (●).

**Figure 4:**
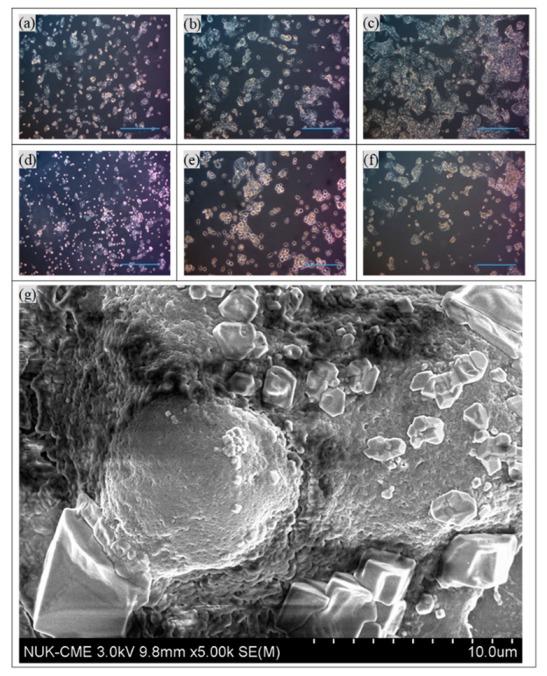
Optical images of HepG2 cells on days 1–3, (**a**)–(**c**), and 2 × 10^4^ cells/well of NK-92 cells added to HepG2 cells on days 1–3, (**d**)–(**f**). Scale bar: 300 μm. (**g**) SEM image of a HepG2 cell with many NK-92 cells.

For the optimization of the ratio NK-92 to HepG2 cells, various cellular concentrations of NK-92 cells were added to HepG2 cells, and the viability of HepG2 cells was measured. The NK-92 cells were in suspension and grew mostly in clumps. In Figure 3a, 10^4^ to 10^5^ NK-92 cells were added to wells containing HepG2 cells. The viability of HepG2 was dramatically reduced, to approximately 40%, for the higher NK-92 numbers. The concentration of NK-92 cells in the human body is 2–72 × 10^4^ cells/mL [29]; thus, 2 × 10^4^ cells/well were used in the subsequent immunotherapy trials. The optical images in Figure 4a–e also confirmed that the concentrations of NK-92 cells increased from 0 to 2 × 10^4^ cells/well in the first three days. To compare the dosage of NPs to HepG2, various concentrations of NPs or MPIP NPs were added to HepG2 or NK-92 cells, respectively, and then the mixtures were added to HepG2 cells. In Figure 3b, the viabilities of HepG2 cells declined dramatically as a result of the activation of NK-92 cells with MPIPs. Please note that zero particles also means zero NK-92 cells in Figure 3b. The most probable mechanistic explanation for this effect is that the PD-1 receptors on the surface of NK-92 cells were blocked by the binding of the MPIPs, causing the cellular viability of the MPIP NPs to be 20% lower than that of the MNIP NPs. Adding MPIP NPs to HepG2, followed by the addition of NK-92 cells, was not quite as effective as when the MPIP NPs were added to the NK-92 cells (presumably blocking them), and the activated cells were then added to the HepG2 cells. However, these results were still significantly better than obtained with MNIP controls, especially at concentrations higher than 200 μg/mL. Figure 4g displays the SEM image of an HepG2 cell that was attacked by NK-92 cells, leaving fragments on its surface.

**Figure 5:**
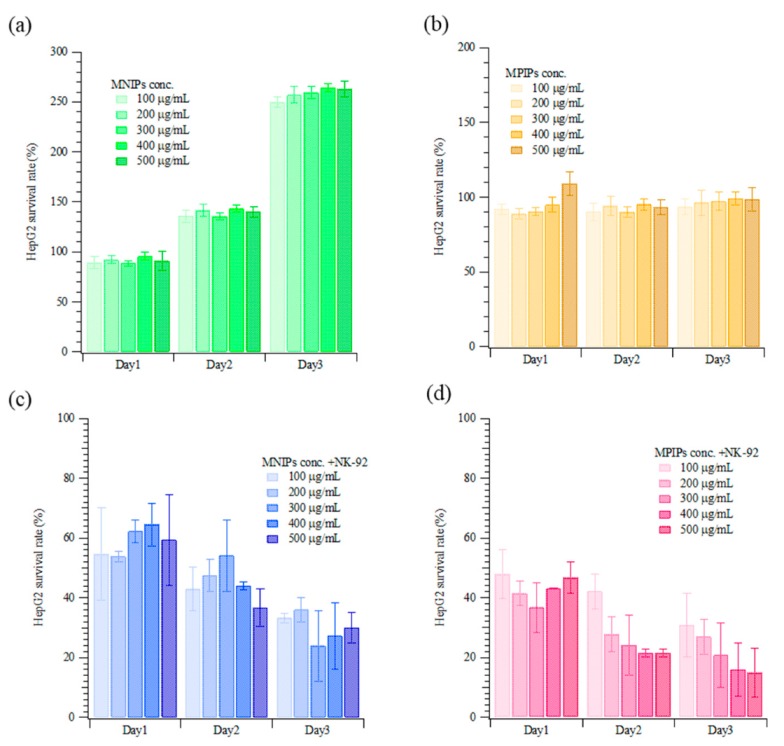
Continuous cellular viability measurements of HepG2 incubated with various concentrations of (**a**) MNIPs, (**b**) MPIPs, (**c**) MNIPs added with NK-92 cells, and (**d**) MPIPs added with NK-92 cells.

Figure 5 displays the results of the treatment of HepG2 cells with nanocarriers and/or NK-92 cells (i.e., MNIPs, MPIPs, MNIPs, or MPIPs with NK-92 cells). HepG2 cell samples were treated daily with specified concentrations of nanocarriers and/or NK-92 cells. In the presence of MNIPs without NK-92 cells, HepG2 cells proliferated well, with 2.5 times as many cells present after three days (Figure 5a). Interestingly, MPIPs alone inhibited the further growth of HepG2 cells (Figure 5b). As expected, treatment with MNIPs and NK-92 cells resulted in significant cell death, with only 24–36% of the cells remaining on day three; this is consistent with the survival expected from treatment with NK-92 cells alone (see Figure 3a). Treatment with MPIPs and NK-92 cells gave the highest mortality, with only 16–30% of the cells remaining on day three (Figure 5c,d).

**Figure 6:**
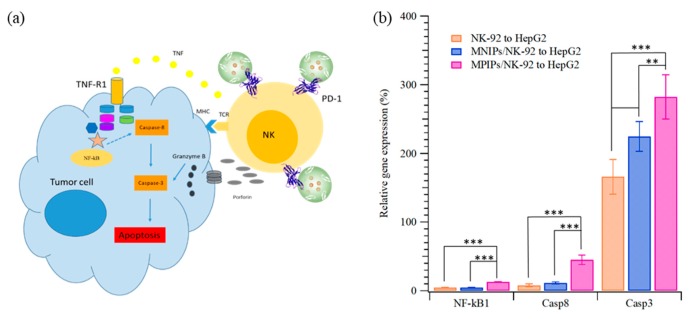
(**a**) Scheme and (**b**) relative expression of nuclear factor kappa B1 (NF-κB1), caspase 8 (Casp8), and Casp3 of HepG2 cells when NK-92, NK-92 with MNIPs, or NK-92 with MPIPs were added (** *p* < 0.005, and *** *p* < 0.0001).

The apoptosis pathway of immunotherapy was enhanced with additional MPIPs/NK-92 cells to promote the expression of NK-κB and then caspase 8 and caspase 3, as shown in Figure 6. The surface protein Fas, an important effector of apoptosis, is a member of the death receptor family in the extrinsic death receptor pathway [30]. Fas is activated by its natural ligand FasL [31]. Nuclear factor kappa B (NF-κB) activity is associated with various cellular events, including proliferation, apoptosis, angiogenesis, and chemo-radioresistance [31]. The expression of NF-κB1 in HepG2 cells was highest when MPIPs with NK-92 cells were added; the expression was much higher than with control MNIPs with NK-92 cells. The Fas-associated protein with death domain (FADD) induces the signaling complex, resulting in apoptotic cell death [32]. Pro-caspase-8 binds bound Fas–FADD to the activated caspase 8 (Casp8) [31], leading to the activation of Casp3 [33]. The increment of NF-κB for MPIPs from MNIPs or NK-92 cells only was about 10%, but that of Casp8 was about 40% from 10%. The expression of Casp3 was 1.7-, 2.2-, and 2.8-fold greater than that on HepG2 cells with NK-92, and MNIPs or MPIPs. These results provide further support for the hypothesis that immunotherapy can be enhanced with the addition of MPIPs, by blocking the PD-1 receptor. It remains possible that MPIPs, but not MNIPs, activate these genes via an unknown mechanism; future studies will be needed to exclude this possibility. Using a magnetic-PD1 peptide-imprinted polymer nanocomposite may allow for future targeted delivery, which could minimize the adverse side effects of conventional PD-1 or PD-L1 inhibitors.

## 4. Conclusions

MPIPs that recognize and bind PD-1 are of great interest because of their potential to inhibit the self-tolerance of natural killer cells and, thus, enahnce immunotherapy for cancer. We synthesized molecularly imprinted nanoparticles, using a peptide derived from PD-1, and showed that these nanoparticles do significantly enhance the activity of natural killer cells toward HepG2 cells. A gene expression analysis revealed increased expression of NF-κB, caspase 8, and especially caspase 3 in the HepG2 cells, especially when treated with NK-92 cells primed with the MPIPs. This work shows the potential of molecularly imprinted nanoparticles in immunotherapy, as natural killer checkpoint inhibitors.

## Figures and Tables

**Table 1:** Prescreening of the binding of peptide molecules to magnetic peptide-imprinted and non-imprinted poly(ethylene-*co*-vinyl alcohol) (EVAL) nanoparticles. The imprinting effectiveness for four different ethylene concentrations (mol.%) is shown. Standard deviations are based on three individual measurements.

EVAL (Ethylene mol.%)	Peptide Adsorption (μg/g)		
	MPIPs	MNIPs	IF
27	34.62 ± 0.26	46.98 ± 1.78	0.74
32	79.60 ± 2.27	33.50 ± 0.14	2.38
38	39.41 ± 0.24	35.03 ± 2.33	1.13
44	50.58 ± 1.39	37.99 ± 0.32	1.33

MPIPs: magnetic peptide-imprinted polymers; MNIP: magnetic non-imprinted polymers; IF: imprinting effectiveness.
